# *CDH3* Retinopathy: Long-Term Multimodal Follow-Up with Pediatric Multidisciplinary Insights

**DOI:** 10.3390/jcm15145393

**Published:** 2026-07-09

**Authors:** Elisa Marziali, Chiavetta Elia, Sara Bargiacchi, Giorgia Mancano, Rosangela Artuso, Elia Dirupo, Lucia Tiberi, Marta Daniotti, Francesca Pochiero, Cesare Filippeschi, Teresa Oranges, Fabiana D’Esposito, Salvatore Angileri, Pina Fortunato, Roberto Caputo, Giacomo Maria Bacci

**Affiliations:** 1 Pediatric Ophthalmology, Meyer Children’s Hospital IRCCS, Viale Pieraccini 24, 50139 Florence, Italy; elisa.marziali@meyer.it (E.M.); eliachiavetta@gmail.com (C.E.); pina.fortunato@meyer.it (P.F.); roberto.caputo@meyer.it (R.C.); 2 Medical Genetics Division, Meyer Children’s Hospital IRCCS, 50139 Florence, Italy; sara.bargiacchi@meyer.it (S.B.); giorgia.mancano@meyer.it (G.M.); rosangela.artuso@meyer.it (R.A.); elia.dirupo@meyer.it (E.D.); lucia.tiberi@meyer.it (L.T.); 3 Metabolic Diseases Unit, Neuroscience Department, Meyer Children’s Hospital IRCCS, 50139 Florence, Italy; marta.daniotti@meyer.it (M.D.); francesca.pochiero@meyer.it (F.P.); 4 Unit of Dermatology, Department of Pediatrics, Meyer Children’s Hospital IRCCS, 50139 Florence, Italy; cesare.filippeschi@meyer.it (C.F.); teresa.oranges@meyer.it (T.O.); 5 Imperial College Ophthalmic Research Group (ICORG) Unit, Imperial College, 153-173 Marylebone Rd., London NW1 5QH, UK; f.desposito@imperial.ac.uk; 6 Department of Neurosciences, Reproductive Sciences and Dentistry, University of Naples Federico II, Via Pansini 5, 80131 Napoli, Italy; 7 Research Collaborator Nurse, Operational Research Unit of Clinical and Cellular Physiopathology, Meyer Children’s Hospital IRCCS, 50139 Florence, Italy; salvatore.angileri@meyer.it

**Keywords:** CDH3, cadherinopathies, pediatric, retinopathy

## Abstract

**Purpose:** To describe the long-term, multimodal follow-up and multidisciplinary evaluation of two pediatric patients with *CDH3*-related retinopathy, discussing the genotypic and phenotypic spectrum of this rare cadherinopathy. **Design:** Retrospective observational case series. **Methods:** Two unrelated children with early-onset macular dystrophy and congenital hypotrichosis underwent a comprehensive ophthalmic examination, including spectral-domain optical coherence tomography (SD-OCT), blue-light fundus autofluorescence (BAF), microperimetry, and full-field electroretinography (ffERG), combined with dermatologic assessment and exome sequencing. Follow-up extended over 4 years in Patient 1 and 15 years in Patient 2. **Results:** Both patients showed sharply demarcated posterior pole chorioretinal atrophy with preservation of the peripheral retina and stable best-corrected visual acuity throughout follow-up. Microperimetry documented localized sensitivity loss with limited progression. SD-OCT revealed persistent ellipsoid zone disruption, retinal pigment epithelium atrophy, and outer retinal tubulations. Dermatologic evaluation confirmed congenital hypotrichosis without nail abnormalities; one patient exhibited mild fifth finger clinodactyly. Genetic testing identified the following two distinct homozygous *CDH3* variants: a splice-site mutation (c.160+1G>A) and a frameshift insertion (c.1837dup, p.(Asp613Glyfs*4)). **Conclusions:** *CDH3* retinopathy presents with a characteristic multimodal imaging pattern of localized macular atrophy and slow functional decline associated with congenital hypotrichosis. A comprehensive multidisciplinary approach was essential for the correct diagnosis and a better and more thorough definition of the clinical presentation. Detailed long-term follow-up supports the hypothesis of retinal pigment epithelium dysfunction or maldevelopment rather than widespread retinal degeneration. Recognition of this phenotype is critical for accurate diagnosis, genetic counseling, and future gene-therapy strategies targeting the preserved peripheral retina.

## 1. Introduction

Hypotrichosis with juvenile macular dystrophy (HJMD, OMIM #601503) is an ultra-rare hereditary disease characterized by alterations in hair growth and distribution and a very early-onset, variably progressive rod–cone retinal dystrophy that affects, almost exclusively, the retinal posterior pole, usually with a fairly symmetrical presentation between the eyes. HJMD is an ultra-rare inherited retinal disorder, with an estimated global prevalence of <1 per 1,000,000 individuals, although precise epidemiological data are limited due to the low number of patients reported so far [1]. This condition usually leads to legal blindness by the second–forth decades of life [2]. This autosomal recessive retinal dystrophy is caused by biallelic pathogenic variants of CDH3 (OMIM #114021), mapped to the long arm of chromosome 16 (16q22.1) [3]. As of today, the pathogenic variants reported in the literature are mostly homozygous, whereas only a few cases are caused by compound heterozygous pathogenic variants [4,5].

HJMD cases are heavily concentrated in communities with higher rates of consanguinity: most of the patients worldwide come from large Turkish, Pakistani, Arab Muslim, and Israeli families living in remote regions of Middle East countries [3,5,6,7,8,9,10,11,12,13,14,15,16,17,18].

The human CDH3 gene spans 54.8 kilobase pairs (kb) across 16 coding exons, and encodes a glycosylated protein called cadherin-3, or P-cadherin (placental–cadherin). Cadherin-3 is part of a superfamily of molecules, called “cadherins”, which plays a crucial role in the development and lifespan of organisms. It consists of five extracellular domains, one transmembrane domain, and one intracellular domain. Cadherin-3 is expressed in hair follicles and RPE (retinal pigment epithelium) cells and is responsible for calcium-dependent cell-to-cell interaction [19].

Variants in CDH3 are also responsible for another even rarer hereditary recessive autosomal disease, called ectodermal dysplasia, ectrodactyly and macular dystrophy (EEM), which combines macular findings typical of those described in HJMD and limb abnormalities [20].

Currently, 95 causative or likely causative variants are reported in the HGMD (Human Genome Mutation Database, https://www.hgmd.cf.ac.uk/ac/index.php (accessed on 7 May 2026)), with 75 associated with retinal disease (72 with HDJM and 5 with EEM). Most of the reported cases lack a detailed phenotype description.

The available literature on HJMD remains limited. To date, most published studies consist primarily of case reports and small case series, reflecting the rarity of this genetic condition. Consequently, the current understanding of its phenotypic spectrum and genotype–phenotype correlations remains incomplete due to fragmented evidence. To provide a clearer overview, existing reports have been systematically reviewed and synthesized in a table summarizing the main genetic findings alongside the key ocular manifestations described in affected individuals (Table 1).

Here, we present two patients with biallelic pathogenic variants in CDH3 showing a complex phenotype. This report aims at highlighting the importance of a multimodal and multidisciplinary approach in such cases that present, nowadays, with a clear genotype–phenotype relationship. A multimodal description of long-term follow-up and extraocular details will be presented for a better understanding of its genotype–phenotype correlations and possible implications for gene-therapy trials.

## 2. Case Description

### 2.1. Patients and Methods

Patient Selection and Ethical Approval. Patients were retrospectively selected from the inherited retinal dystrophy database of the Meyer Children’s Hospital (Florence, Italy) covering a period from 2000 to the present. Inclusion criteria required a conclusive molecular diagnosis of CDH3 pathogenic variants and a well-established genotype–phenotype correlation. The study adhered to the tenets of the Declaration of Helsinki and was formally approved by the local Institutional Ethics Committee (Protocol Code: PA_03/2025). Written informed consent was obtained from the parents or legal guardians of all minors prior to data collection.

Data Collection and Ophthalmic Evaluation Medical charts were meticulously reviewed to ensure maximum data integrity. Longitudinal clinical data extracted included demographics and a complete ophthalmological evaluation, such as at least best-corrected visual acuity (BCVA), color vision testing, and findings from both anterior segment and dilated fundus examinations.

Follow-up Schedules, Imaging, and Functional Testing Protocols. Patients underwent annual follow-up visits, during which comprehensive multimodal imaging was systematically performed to monitor structural changes. Morphological evaluation included spectral-domain optical coherence tomography (SD-OCT), initially acquired with the Optovue iVue (Optovue Inc., Fremont, CA, USA) and subsequently with the Spectralis OCT Duo (Heidelberg Engineering, Heidelberg, Germany). Fundus autofluorescence (FAF) was captured using the Spectralis platform, and color fundus photography was performed using a Topcon fundus camera (Topcon Corp., Tokyo, Japan).

Additional functional testing was performed periodically during the annual follow-ups, strictly contingent upon the patients reaching an adequate level of cooperation. When sufficient compliance was achieved, macular sensitivity was assessed via microperimetry utilizing the Macular Integrity Assessment (MAIA) microperimeter (CenterVue, Padova, Italy) under standard testing conditions. Furthermore, full-field electroretinography (ffERG) was recorded using a CSO system (Costruzione Strumenti Oftalmici, Florence, Italy) in accordance with the International Society for Clinical Electrophysiology of Vision (ISCEV) guidelines.

*Patient 1*. A 10-years-old Caucasian male of Albanian origin was referred to the pediatric inherited eye disease clinic for bilateral vision loss. The patient exhibited a distinctive appearance with thin and sparse hair. He is the only child of non-consanguineous parents. His family history includes a maternal aunt with a not further specified unilateral eye disease, diagnosed at age 30 but reported to have been present since birth.

**Figure 1:**
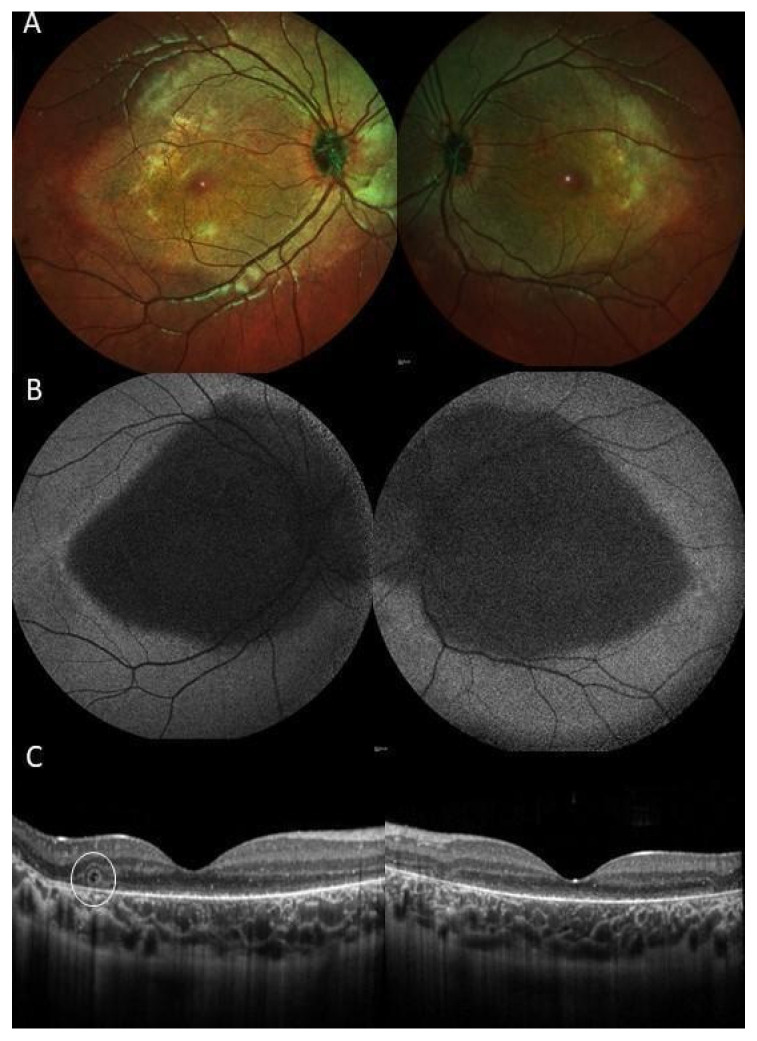
(**A**) color fundus imaging; (**B**) blue autofluorescence imaging; (**C**) structural OCT examination of Patient 1 at the baseline. (**A**) Color fundus examination and blue fundus imaging showed an area of chorioretinal atrophy and pigmentary changes involving the entire posterior pole within the vascular arcades. (**B**) Blue fundus autofluorescence imaging demonstrated an extensive area of hypoautofluorescence at the posterior pole, while the peripheral retina showed no detectable abnormalities. (**C**) Structural OCT examination showing abnormalities at the level of the outer retinal layers. Outer retinal tubulation was also detectable in the right eye as an ovoid hyporeflective structures with hyperreflective outer walls, located within the outer nuclear/photoreceptor layers (white circle).

At the first ophthalmological evaluation, his best-corrected visual acuity (BCVA) was 0.4 LogMAR in both eyes. He never reported nyctalopia or photophobia. The saturated panel D-15 color test was normal. Anterior segments and intraocular pressure were unremarkable. A fundus dilated examination showed bilateral appearance of retinal dystrophic changes at the posterior pole that extended toward the arcades sharply sparing the mid and far periphery (Figure 1). Blue light-autofluorescence (B-AF) examination revealed a corresponding area of hypoautofluorescence (Figure 1). Fundus autofluorescence imaging did not reveal abnormal hyperautofluorescent lesions or other findings suggestive of lipofuscin accumulation in any of the examined patients.

Structural macular optical coherence tomography (SD-OCT, Spectralis, Heidelberg Engineering, Heidelberg, Germany) examination showed diffuse abnormalities of the ellipsoid zone (EZ) and RPE atrophy in both eyes. Moreover, outer retinal tubulations (ORTs) were detectable on the right (Figure 1). Microperimetry (MAIA, Centervue, Padua, Italy) showed diffuse abnormal sensitivity within the dystrophic area. Interestingly, a sharp anatomic and functional demarcation line was evident in the transitional area between normal and pathological retinas.

### 2.2. Dermatological Features

**Figure 2:**
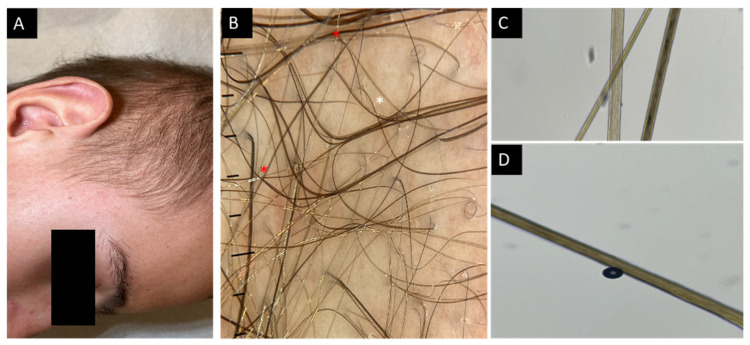
(**A**) showing sparse hair in a 10-year-old child with HJMD; (**B**) Trichoscopic examination shows variations in hair-shaft thickness, coiled hairs (red asterisks), and circle hairs (white asterisks). Microscopic examination shows variations in hair-shaft thickness (**C**) and flattening along a hair shaft (**D**).

In Patient 1, we also performed a dedicated multidisciplinary evaluation that highlighted significant dermatological findings; more specifically, dermatologists revealed a form of hypotrichosis, as the patient showed sparse hair (Figure 2A), with neither eyebrow abnormalities nor dysplastic nails. Trichoscopy (Figure 2B) showed variations in hair shaft thickness and multiple coiled and circle hairs. Upon microscopic examination (Figure 2C,D), flattening along hair shafts was also observed. A clinical diagnosis of HJMD was confirmed by genetic analysis. Given the presence of bilateral V finger clinodactyly, an EEM diagnosis could be taken into account as well.

**Figure 3:**
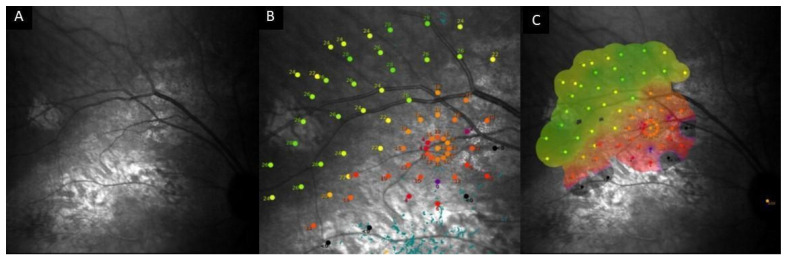
(**A**) Infrared imaging of the area within the healthy and the affected retina, the so-called transition zone; (**B**) quantitative and (**C**) qualitative microperimetry findings at the transition zone in Patient P2. The microperimetry findings revealed sharp impairment of retinal sensitivity in this area: brighter colors indicate better retinal sensitivity, while darker colors indicate impaired retinal sensitivity.

**Figure 4:**
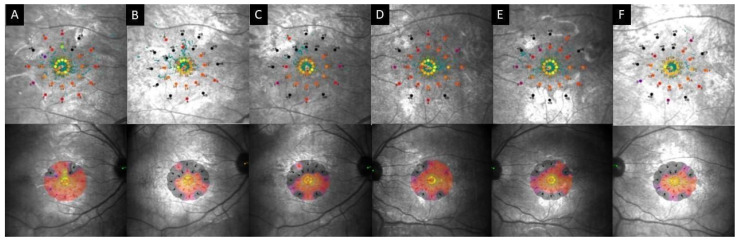
Infrared imaging and microperimetry assessment over the follow-up: longitudinal microperimetry findings of Patient P1 during follow-up in the (**A**–**C**) right and (**D**–**F**) left eye, respectively. Colorimetrics note: darker tones indicate reduced retinal sensitivity, lighter tones indicate better retinal sensitivity.

During the 4-year-old’s follow-up, the patient’s visual acuity remained stable, with no major anatomical changes. A functional progression of the disease was detected only through microperimetry testing (Figure 3 and Figure 4).

*Patient 2*. A 3-year-old Italian female, living with adoptive parents, was referred to the pediatric inherited eye disease clinic for a suspected retinal dystrophy associated with bilateral visual loss and strabismus; no nyctalopia or photophobia were reported. Her personal history was unremarkable, although the family reported sparse hair from her early infancy. The patient’s follow-up was conducted using a multimodal imaging approach including structural OCT, color fundus, blue light-autofluorescence (BAF), full-field electroretinography (ff-ERG), and visual evoked potentials (VEPs). Brain MRI was also performed during the initial assessment.

At first ophthalmological evaluation, her BCVA was 0.7 LogMAR in both eyes with mild hyperopia and moderate astigmatism. Saturated Panel D-15 showed an aspecific abnormal pattern. The anterior segment was unremarkable. Fundus examination revealed dystrophic retinal changes spreading from the posterior pole toward the arcades with a sharp restore of normal retinal appearance in the peripheral retina (Figure 4).

**Figure 5:**
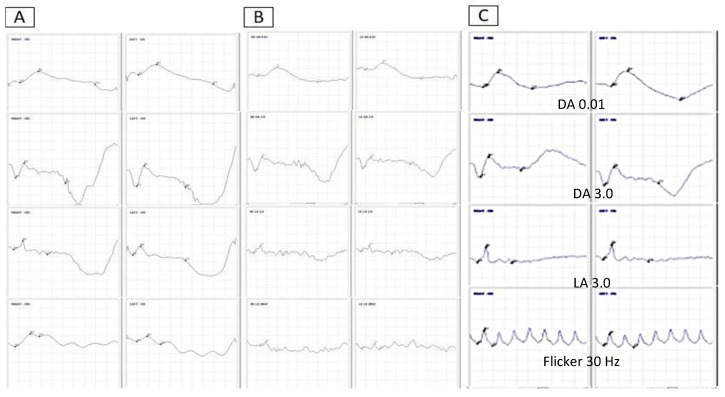
Full-field electroretinography (ff-ERG) findings in Patient P2 remained stable between baseline evaluation in 2016 (**A**) and follow-up assessment after 7 years in 2023 (**B**). (**C**) A normal full-field electroretinogram (ff-ERG).

Structural SD-OCT examination at the macula showed ellipsoid zone (EZ) loss and RPE atrophy. A diffuse thinning of the inner retinal layers was also detectable (Figure 5).

### 2.3. Summary

Overall, both patients demonstrated consistent structural and functional stability throughout the follow-up period. This stability was confirmed by longitudinal best-corrected visual acuity (BCVA) measurements, which remained unchanged, as well as by stable full-field electroretinography (ffERG) data. Central retinal thickness on optical coherence tomography (OCT) scans remained stable as well: in the female patient (Supplemental Figure S1), measurements were 206 µm (RE) and 210 µm (LE) in 2017, compared to 199 µm (RE) in 2024 (LE data unavailable). Similarly, the male patient (Supplemental Figure S2) showed measurements of 187 µm (RE) and 214 µm (LE) in 2021 and 226 µm (RE) and 236 µm (LE) in 2024. Despite minor changes in the outer retinal layers, these findings indicate preserved structural integrity over time.

Electroretinography recorded with skin electrodes were conducted at 3 and 5 years of age in a different hospital and showed no progressive reductions in photopic and scotopic responses. A more recent full-field electroretinogram (ffERG) was performed according to ISCEV standard: at 9 years, both eyes showed preserved scotopic and reduced photopic responses. The patient underwent a further ffERG at age 17 showing the same results (Figure 5). Visual evoked potentials were performed firstly at the age of 5 at a different eye clinics and showed severe reduction in N75-P100 wave amplitude and prolonged latency. To rule out any other associated condition, we conducted a brain MRI that showed normal findings.

**Figure 6:**
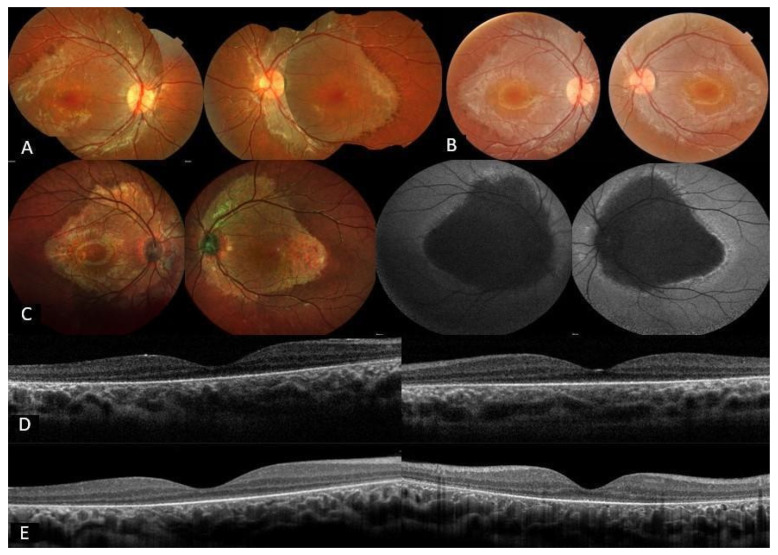
Multimodal imaging of Patient P2: (**A**) Color fundus photography at the first follow-up visits in 2012, (**B**) during follow-up and (**C**) at the last follow-up visit in 2021. (**A**–**C**) Fundus imaging demonstrated chorioretinal atrophy involving the entire posterior pole sparing the peripheral retina. The optic nerve head appeared healthy, and retinal vessels were of normal caliber. These findings remained stable throughout the follow-up period. Fundus autofluorescence imaging showed an hypoautofluorescent area covering the entire posterior pole corresponding to the area of chorioretinal atrophy detactable on color fundus imaging. The area of atrophy extends to the optic nerve, encircling it (**D**,**E**): structural OCT examination performed in (**D**) 2017 and (**E**) 2019 in both eyes, respectively. OCT scan at the macula showed foveal photoreceptor rarefaction. The ellipsoid zone was impaired at the fovea in both eyes. Moreover, a thinning of the outer nuclear layer (ONL) was also detectable. OCT scans acquired in 2017 (**D**) and 2019 (**E**) demonstrated marked morphological stability.

BCVA remained stable in both eyes during the 15-year follow-up, while only minor changes were detectable with SD-OCT (Figure 6).

Multidisciplinary evaluation was performed. Given the co-occurrence of retinal dystrophy and hypotrichosis, CDH3 involvement was suspected and subsequently confirmed by genetic testing.

## 3. Genotyping Analysis

Exome Sequencing sequencing (ES) was performed on probands using DNA extracted from peripheral blood by standard methods. The genomic DNA was sequenced with the platform NextSeq500/550 (Illumina Inc., San Diego, CA, USA); reads were aligned to the human reference hg19 genome using Burrows–Wheeler Aligner (v 0.7.12) [33]. Downstream alignment processing (i.e sorting, indexing, deduplication, and base quality score recalibration) was performed with the Genome Analysis Toolkit (GATK) (v 3.7) [34], SAMtools (v 1.16.1) [33], and Picard Tools (https://broadinstitute.github.io/picard/, accessed on 7 May 2026) (v.1.119). GATK HaplotypeCaller was used to obtain a set of single nucleotide variants and indel calls for genes associated to retinal dystrophy. Variant prioritization and interpretation was performed using workflow previously described in Rocca et al. (2022) [35].

In Patient 1, an apparently homozygous pathogenic variant in the splicing donor site of intron 2 of CDH3 (NM_001793.6), c.160+1G>A, was found. As the father was unavailable, only the mother could be tested, and she was found to be heterozygous. According to ACMG (American College of Medical Genetics and Genomics) guidelines, this variant is classified as pathogenic (PVS1, PP5, PP4, and PM2), and it is predicted to induce a large splicing change [36].

In Patient 2, an apparently homozygous (the patient being adopted, her biological parents were unavailable for testing) pathogenic variant, c.1837dup, (p.(Asp613Glyfs*4)), of CDH3 (NM_001793.6) was found, which had already been reported in the literature [37] as a variant of unknown significance (VUS). According to ACMG guidelines, this variant is now classified as pathogenic (PVS1, PM2, and PP5). In addition to HGMD, we reviewed population frequency data available in gnomAD. The variant is extremely rare in our internal database and in the general population, being reported with an allele count of 1 and with no homozygous individuals identified in the database. Furthermore, ClinVar (rs1157340043) currently includes two independent submissions classifying this variant as pathogenic. These observations confirm the pathogenicity of this variant.

## 4. Discussion

CDH3 encodes P-cadherin, which belongs to a group of calcium-dependent intercellular adhesion molecules well-known to be associated with inherited retinal degeneration encompassing both developmental and degenerative disorders [19].

Our study provides a detailed phenotypic and genotypic analysis of two patients with confirmed *CDH3*-related disease, highlighting both ophthalmic and systemic findings. The description of these two pediatric patients confirms that pathogenic variants in CDH3 are related to an extremely peculiar phenotype characterized by early onset retinal dystrophy along with distinctive extraocular signs such as congenital hypotrichosis. The novelty of our work lies in the comprehensive phenotypic description over a mid- and long-term follow-up (4 and 15 years, respectively).

There is a lack of retinal imaging data for patients with *CDH3*-related retinal dystrophies in the scientific literature. Hull et al. [22] reported the largest series of HJMD patients describing their clinical presentation and disease progression. Altogether, these data indicate that *CDH3*-related retinopathy typically manifests in childhood with progressive chorioretinal atrophy. Interestingly, this condition can be differentiated from other juvenile macular dystrophies by the presence of congenital hypotrichosis, with some patients also exhibiting limb abnormalities. Until now, no phenotype–genotype correlations have been established, likely due to the extensive heterogeneity observed in CDH3 patients among scientific reports. Although P-cadherin is known to be expressed in the follicles and in the RPE genesis, as well as being potentially implicated in melanocyte development [9], its precise role in RPE survival is yet to be completely understood.

Another key point is the fact that *CDH3*-associated juvenile macular dystrophy (HJMD-like) is highly likely an RPE-driven entity rather than a primary retinal developmental dysgenesis; P-cadherin is a key cadherin in the RPE, and clinical cohorts describe macular disease dominated by outer retinal/RPE atrophy and frequent outer retinal tubulation (ORT) on OCT, a remodeling feature commonly linked to photoreceptor degeneration in the setting of RPE instability/atrophy [22,38]. “Developmental” cadherinopathy arguments remain speculative for the retina and are supported mainly by the syndromic context (congenital hypotrichosis ± ectodermal/limb findings) and occasional reports proposing shared pathways between ectodermal development and retinal degeneration (e.g., cone–rod dystrophy presentations) [25].

Recently, Yussuf et al. published a review on retinal “cadherinopathies” and suggested a possible, viable target for therapeutic approaches; although not yet fully confirmed, the actual localized pattern of this peculiar disease, mainly affecting central vision, coupled with the relatively limited progressivity, could have major potential therapeutic implications, since subretinal gene therapy could be the best way to preserve and/or restore retinal function, in a context of unaffected retinal periphery [19].

This report expands the description of patients with CDH3 mutations and thoroughly describes the multidisciplinary approach to ophthalmic, genetics and dermatological features. Moreover, we report a new patient with the recurrent c.160+1G>A CDH3 variant, and we confirm pathogenicity for another variant in an ultra-rare EEM mild phenotype. We must recognize that this study has severe limitations; although rare, the report of two cases is insufficient to draw any conclusions for possible genotype–phenotype correlations; however, it can surely prompt some considerations on structure–function stemming from the observation of the phenotypes. Moreover, since clinical electrophysiology testing in our patients is limited to ff-ERG, we cannot exclude that a more specific electrophysiology test (i.e., mf-ERG) and a longer term follow-up could detect a progression of the condition in both central retinal function and peripheral progression of the disease.

## 5. Conclusions

*CDH3* retinal dystrophy is today a well-known cause of cadherin-related retinal dystrophy with potential for future gene-therapy trials. With a deep multimodal, multidisciplinary and long-term follow-up, this report provides knowledge for clinicians to manage this peculiar retinal dystrophy and give proper counseling to affected families. Since only a few papers exist in the literature, mainly case reports or case series, we think that a future meta-analysis of published studies could be a feasible way to shed more light on the natural history and target.

## Figures and Tables

**Table 1:** Literature review of clinical characteristics and mutations identified in the CDH3 gene in patients with HJMD.

Reference	Origin of Patients	Visual Acuity (OD/OS)	Scalp Hypo- trichosis	Macular Pigment Degeneration	Follow-Up (Years)	Number of Patients in a Family	Additional Clinical Findings	CDH3 Variants (NM_001793.6) and Zygosity
Sprecher et al. (2001) [3]	Israeli	N/A	YES	YES	N/A	11	N/A	c.981del p.(M327Ifs*23), homozygous
Indelman et al. (2002) [6]	Israeli	N/A	YES	YES	N/A	4	N/A	c.1508G>A p.(R503H), homozygous
Indelman et al. (2003) [7]	French	N/A	YES	YES	N/A	N/A	Atopic Dermatitis	c.503T>A p.(L168*), heterozygous c.2112del p.(G706Vfs*53), heterozygous
Indelman et al. (2003) [7]	Turkish	N/A	YES	YES	N/A	N/A	Keratosis pilaris	c.829del p.(G277Afs*20), homozygous
Indelman et al. (2003) [7]	Israeli	N/A	YES	YES	N/A	N/A	Centrofacial lentiginosis	c.1508G>A p.(R503H), homozygous
Indelman et al. (2003) [7]	Israeli	N/A	YES	YES	N/A	N/A	N/Ains	c.462del p.(E155Rfs*6), homozygous
Bergman et al. (2004) [8]	Israeli	N/A	YES	YES	N/A	N/A	N/A	c.1508G>A p.(R503H), homozygous
Indelman et al. (2005) [9]	Arab	p1 RE 0.1, LE 0.1 p2 N/A	YES	YES	N/A	2	N/A	c.1845T>G p.(Y615*), homozygous
Leibu et al. (2006) [10]	Israeli	N/A	YES	YES	N/A	2	N/A	c.981del p.(M327Ifs*23), homozygous
Indelman et al. (2007) [5]	American	N/A	YES	YES	N/A	3	Discolored primary teeth, nail dystrophy	c.661C>T p.(R221*), heterozygous c.1724A>G p.(H575R), heterozygous
Indelman et al. (2007) [5]	English	RE:0.8, LE: 0.1	YES	YES	N/A	1	Limb abnormalities	c. 160+1G>A p.?, heterozygous c.1510G>A p.(E504K), heterozygous
Jelani et al. (2009) [11]	Pakistani	N/A	YES	YES	N/A	13	N/A	c.1425−1G>T p.?, homozygous
Kamran-ul-Hassan Naqvi et al. (2010) [12]	Pakistani	N/A	YES	YES	N/A	6	N/A	c.1425−1G>A p.?, homozygous
Shimomura (2010) [13]	Pakistani	N/A	YES	N/A	N/A	4	N/A	c.1796−2A>G p.?, homozygous
Shimomura et al. (2010) [13]	Pakistani	N/A	YES	N/A	N/A	3	N/A	c.1425−1G>T p.?, homozygous
Avitan-Hersh, Indelman, Khamaysi, Leibu, and Bergman (2012) [14]	Arab	N/A	YES	YES	N/A	1	N/A	c.747C>A p.(Y249*), homozygous
Halford, Holt, Nemeth, and Downes (2012) [15]	N/A	RE 2.0, LE 1.2	YES	YES	N/A	1	N/A	deletion causing skipping of exons 12–13 of *CDH3*, homozygous
Khan and Bolz (2016) [16]	Arab	RE 0.5, LE 0.5	YES	YES	N/A	2	Slow nail growth	c.307C>T p.(R103*), homozygous
Khan and Bolz (2016) [16]	Arab	OU 0.16	YES	YES	N/A	2	N/A	c.1859_1862delCTCT p.(S620Cfs*10), homozygous
Singh et al. (2016) [21]	N/A	RE 0.63; LE 0.80 than RE 0.25 LE 0.5	YES	YES	3	1	N/A	c.1796-2A>G p.?, homozygous
Hull (2016) [22]	German	OU 0.32 than 0.63	YES	YES	1	1	N/A	c.316_317delAA p.(K106Efs*12) heterozygous, c.1086G>A p.(W362*), heterozygous
Hull (2016) [22]	Pakistani	RE 0.5, LE 0.8 than RE 0.25 OS CF	YES	YES	2	3	N/A	c.1425-1G>T p.?, homozygous
Hull (2016) [22]	Pakistani	OU 0.4 than RE 0.16 LE CF	YES	YES	6	1	N/A	c.1568delA p.(N523Mfs*14), homozygous
Hull (2016) [22]	Jordan	RE 0.10 LE 0.06	YES	YES	N/A	1	N/A	c.1508G>A p.(R503H), homozygous
Hull (2016) [22]	Turkish	OU 0.8 than RE 0.8 LE 0.3	YES	YES	8	1	N/A	c.829del p.(G277Afs*20), homozygous
Hull (2016) [22]	Portugal	RE 0.10 LE 0.06	YES	YES	N/A	1	N/A	c.613G>A p.(V205M), homozygous
Hull (2016) [22]	Portugal	OU 0.63	YES	YES	20	1	N/A	c.829del p.(G277Afs*20), homozygous
Hull (2016) [22]	Pakistani	RE 0.25 LE 0.08 than RE 0.16LE 0.06	YES	YES	3	2	N/A	c.2357delG p.(G786Afs*7), homozygous
Hull (2016) [22]	Pakistani	OU HM than HM	YES	YES	3	3	N/A	c.2157C>T p.(R720*), homozygous
Hull (2016) [22]	English	OU 1.25 than OU 0.06	YES	YES	7	1	N/A	c.160+1G>A p.?, homozygous
Karti et al. (2017) [23]	Turkish	RE 0.9, LE 0.1	YES	YES	N/A	1	N/A	c.447_467del p.(A150_G156del), homozygous
Blanco-Kelly et al. (2017) [24]	Spanish	RE 0.08, LE 0.1 than OU 0.15	YES	YES	8	1	N/A	c.830delG p.(G277Afs*20), homozygous
Vicente et al. (2017) [2]	Iranian	OU 0.5	YES	YES	N/A	1	Mild eczema, a missing left index fingernail	c.640A>T p.(K214*), homozygous
Nasser et al. (2019) [25]	Syrian	RE 0.20, LE 0.40	YES	YES	N/A	1	Hypoplastic nails	c.1508G>A p.(R503H), homozygous
Saeidian (2019) [26]	Iranian	OU 1.00 than OU 0.05	YES	YES	N/A	10	Hypoplastic nails	deletion causing skipping of exon 3 of *CDH3*, homozygous
Oliveira-Ferreira (2019) [27]	Caucasian	RE 1.25, LE 0.5	YES	YES	N/A	1	N/A	c.830delG p.(G277Afs*20), homozygous
Schauren (2019) [28]	Brazilian	N/A	YES	YES	N/A	N/A	N/A	c.160+1G>A p.?, homozygous
Schauren (2019) [28]	Brazilian	N/A	YES	YES	N/A	N/A	N/A	c.160+1G>A p.?, heterozygous c.1063G>T p.(D355Y), heterozygous
Schauren (2019) [28]	Brazilian	N/A	YES	YES	N/A	N/A	N/A	c.1795+1G>C p.?, homozygous
Narayan (2019) [29]	Russian	RE 0.32 LE 0.40	YES	YES	N/A	1	N/A	c.1508G>A p.(R503H), homozygous
Narayan (2019) [29]	Russian	RE 0.8 LE 0.5	YES	YES	N/A	1	N/A	c.1508G>A p.(R503H), homozygous
Nasser (2020) [30]	German	RE 0.10; LE 0.40	YES	YES	N/A	1	N/A	c.1508G>A p.(R503H), homozygous
Nasser (2020) [30]	German	RE 0.50 LE 0.25	YES	YES	N/A	1	N/A	c.1508G>A p.(R503H), homozygous
Hayashi (2021) [4]	Japanese	OU 0.8, then OU 0.7; RE 0.1 LE 0.8 then RE 0.07 LE 0.5	YES	YES	5	2	n/A	c.123_129dupAGGCGCG (p.E44fs*26), heterozygous c.2280+1G>T p.?, heterozygous
Ahmed (2021) [31]	Arabian	OU 0.05	YES	YES	N/A	2	N/A	c.C307T p.(R103*), homozygous
Prieto (2025) [32]	Columbian	N/A	YES	YES	N/A	1	Severe micrognathia	c.1508G>A p.(R503H), homozygous

## Data Availability

The raw data supporting the conclusions of this article will be made available by the authors on reasonable request.

## Supplementary Materials

The following supporting information can be downloaded at: https://www.mdpi.com/article/10.3390/jcm15145393/s1, Figure S1: Longitudinal OCT scans of macular morphology of Patient 2; Figure S2: longitudinal OCT scan of macular morphology of Patient 1.
