# Supplementary figures and images for: CDH3 Retinopathy: Long-Term Multimodal Follow-Up with Pediatric Multidisciplinary Insights
Source: J Clin Med. 2026 Jul 9;15(14):5393. doi: 10.3390/jcm15145393 (PMC13412677; doi:10.3390/jcm15145393)

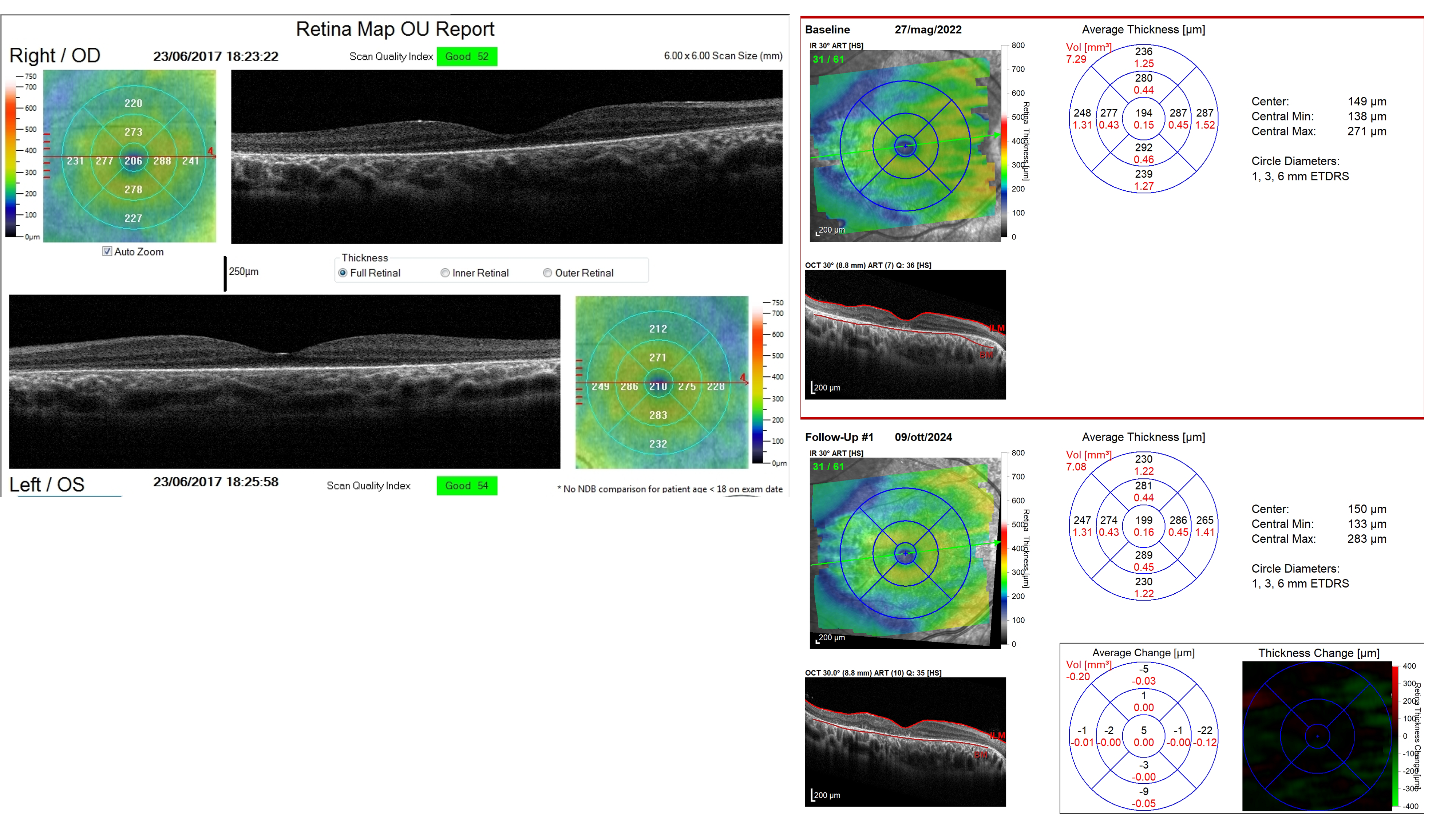

Supplement: Supplementary file 1 [file jcm-15-05393-s001.zip › Supplemental Figure S1.tiff]

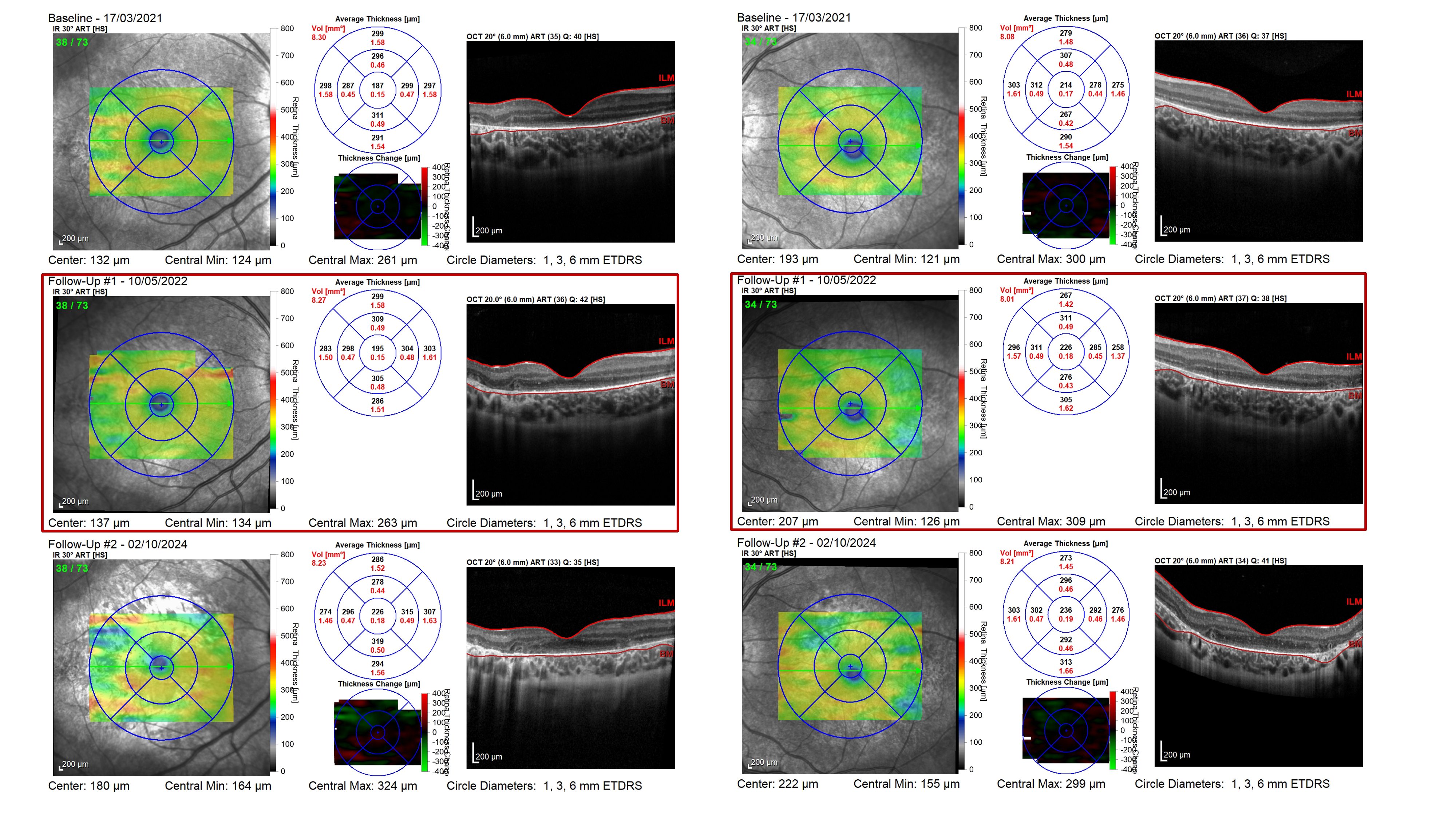

Supplement: Supplementary file 1 [file jcm-15-05393-s001.zip › Supplemental Figure S2.tiff]
